# Rapid adaptive responses of rosette‐type macrophyte *Vallisneria natans* juveniles to varying water depths: The role of leaf trait plasticity

**DOI:** 10.1002/ece3.8142

**Published:** 2021-09-22

**Authors:** Yuxuan Gao, Lei Wang, Xiaoqing Hu, Zhuolun Zhang, Baogui Liu, Xinhou Zhang, Guoxiang Wang

**Affiliations:** ^1^ Jiangsu Engineering Lab of Water and Soil Eco‐remediation School of Environment Nanjing Normal University Nanjing China

**Keywords:** functional traits, phenotypic plasticity, shallow lake, submerged macrophyte, water depth

## Abstract

Rosette‐type submerged macrophytes are widely distributed across a range of water depths in shallow lakes and play a key role in maintaining ecosystem structures and functions. However, little is known about the rapid adaptive responses of such macrophytes to variations in water depth, especially at the juvenile stage. Here, we conducted a short‐term in situ mesocosm experiment, in which the juveniles of *Vallisneria natans* were exposed to a water depth gradient ranging from 20 to 360 cm. Twenty‐two leaf‐related traits were examined after 4 weeks of growth in a shallow lake. Most (18) traits of *V. natans* generally showed high plasticity in relation to water depth. Specifically, juveniles allocated more biomass to leaves and had higher specific leaf area, leaf length‐to‐width ratio, chlorophyll content, and carotenoids content in deep waters, displaying trait syndrome associated with high resource acquisition. In contrast, *V. natans* juveniles in shallow waters had higher leaf dry matter content, leaf soluble carbohydrate content, carotenoids per unit chlorophyll, and peroxidase activity, pertaining to resource conservation. Notably, underwater light intensity was found to be the key factor explaining the trait plasticity along the water depth gradient, and 1.30 mol photons m^−2^ d^−1^ (at 270 cm) could be the optimal irradiance level based on the total biomass of *V. natans* juveniles. The present study highlights the significance of leaf trait plasticity for rosette‐type macrophytes in response to variations in water depth and sheds new light on the differences between trade‐offs in deep‐ and shallow‐water areas.

## Graphical abstract

Rosette‐type submerged macrophytes play a key role in maintaining ecosystem structures and functions. The present study highlights the significance of leaf trait plasticity for rosette‐type macrophytes in response to variations in water depth and sheds new light on the differences between trade‐offs in deep‐ and shallow‐water areas.

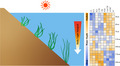

## 1 INTRODUCTION

In shallow lakes, submerged macrophytes are the key ecosystem engineers maintaining ecosystem structure and functioning (Jeppesen et al., 2012; Scheffer et al., 2001). As primary producer, macrophytes provide food and refuge for fish and zooplankton and serve as a habitat for periphyton (Burks et al., 2001; Jeppesen et al., 1997; Roberts et al., 2003). Dense submerged macrophytes effectively improve the physical and chemical environment by stabilizing the sediment, absorbing nutrients, and releasing oxygen (Liu et al., 2020; Lürig et al., 2020; Madsen et al., 2001). Moreover, the allelopathic activity of several macrophyte species could inhibit the growth of phytoplankton (van Donk & van de Bund, 2002). Consequently, submerged macrophytes play a key role in enhancing water clarity and maintaining shallow lakes in the clear water state (Scheffer et al., 1993; Søndergaard et al., 2007).

Variations in water depth greatly impact the growth and development of submerged macrophytes (Coops et al., 2003; Ersoy et al., 2020), due to changes in multiple environmental factors, especially the underwater light intensity (Wang, Wang, et al., 2021; Yuan et al., 2018). Behaving like shade‐adapted species, submerged macrophytes generally have a low tolerance of high light intensity in shallow‐water environments (Bowes & Salvucci, 1989; Hussner et al., 2010), whereas reduced light availability in deep waters can hamper their photosynthesis or root respiration (Han et al., 2019). The optimal water depth range has been identified for numerous species (Liu et al., 2016), outside of these ranges, macrophytes have to make adjustments. For canopy‐forming macrophytes (e.g., *Potamogeton maackianus* and *Hydrilla verticillata*), it is well established that stem elongation toward the water surface with increasing water depths is the key adaptive response to counter light shortage (He et al., 2019). In contrast, rosette‐type macrophytes have been less well studied, though which are common species, at a series of water depths in shallow lakes (e.g., *Vallisneria* spp., grow at 20–200 cm depth in east China, see Zhou et al., 2016). Therefore, identification of the rapid adaptive responses that rosette‐type macrophytes make to varying water depth is urgently needed. This knowledge could help reveal the mechanisms behind macrophyte distribution, but also guide ecological restoration of degraded systems. Furthermore, information regarding the juvenile stage is especially important, as the establishment of juveniles (from seeds, tubers, or stolons) plays a crucial role in constructing macrophyte communities and subsequently against the phytoplankton bloom.

In recent decades, extreme climate events, such as extreme precipitation, droughts, and heat waves, have led to frequent occurrence of unusual water depths in lakes (Woolway et al., 2021) and subsequently disappearance of submerged macrophytes. For example, widespread loss of submerged vegetation in Lake Okeechobee (Florida) was believed to result from the above‐average water depth during 1994 to early 2000, as well as the extensive drought in summer 2001 (Harwell & Havens, 2003). Unfortunately, no empirical studies have been performed to test the performance of rosette‐type macrophytes in extremely shallow (or deep) water scenarios, though such knowledge is of great value for predicting the stability of macrophyte communities in response to extreme hydrology.

As sessile organisms, plants have evolved mechanisms to adaptively response to environmental changes by modifying their morphological and physiological traits (Nicotra et al., 2010; Pazzaglia et al., 2021), as well as the biomass allocation (Shipley & Meziane, 2002). Leaf traits and trait syndromes (consistent associations of multiple traits) are closely correlated with the trade‐off between resource acquisition and resource conservation (Annighöfer et al., 2017; Dalla Vecchia et al., 2020; Meng et al., 2020; Wright et al., 2004). Plant individuals that are resource acquisitive usually share similar attributes such as larger leaves and higher specific leaf area, while those resource conservative ones generally carry a higher leaf density and greater leaf dry matter content (Adler et al., 2014; Reich & Cornelissen, 2014; Wright et al., 2004). For rosette‐type macrophytes without stems, the plasticity of leaf traits may play a particularly important role in reflecting their rapid adaptive responses to vary water depths.

*Vallisneria* spp., typical rosette‐type macrophytes, are widely distributed in freshwater ecosystems located in East Asia, North America, South Europe, and Australia (Biernacki & Lovett‐Doust, 1997; Les et al., 2008; Lowden, 1982; Shen et al., 2021). Moreover, *Vallisneria* planting has been extensively used in the ecological restoration of degraded lakes (Gao et al., 2017). However, relatively little is known about the leaf trait plasticity and adaptive performance of *Vallisneria* at the juvenile stage. Based on an in situ mesocosm experiment, we determined growth and allocation traits, leaf morphology and anatomy traits, and leaf physiology traits of juvenile *Vallisneria natans* grown at water depths ranging from 20 to 360 cm in a shallow lake environment. We specifically aimed at testing the hypotheses that juvenile *V. natans* can rapidly response to a wide range of water depths through trait plasticity, displaying traits associated with higher resource acquisition in deep waters to manage a shortage of light, and tending to resource conservation in extremely shallow water to withstand excessive light intensity.

## 2 MATERIALS AND METHODS

### 2.1 Study site

**FIGURE A1:**
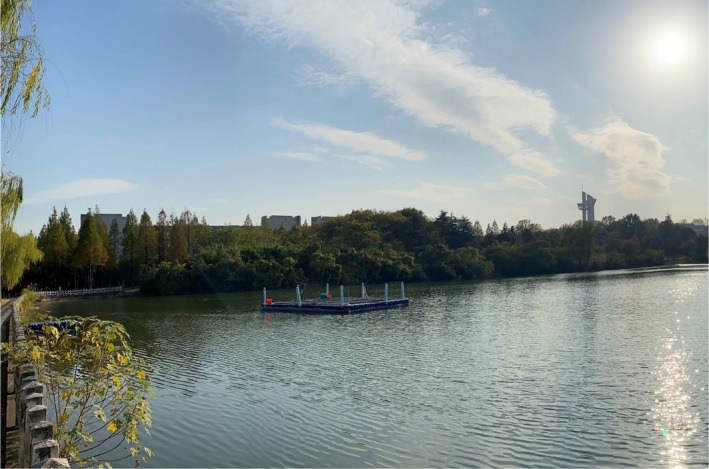
View of the study site

The in situ mesocosm experiment was carried out in Caiyue Lake (32°10′N, 118°91′E), a macrophyte‐dominated lake located in Jiangsu Province, Eastern China. This lake experiences a subtropical monsoon climate, with a annual mean air temperature of 17.5℃ and mean annual precipitation of 1,421 mm (2016–2020). The lake is characterized by an average water depth of 1.8 m, a maximum depth of 4.5 m, and a water surface area of 21,000 m^2^. In October 2019, a floating platform (8 × 8 m) was assembled and anchored on the eastern side of the lake (Figure A1), where the average water depth was approximately 4 m (Secchi depth: 150–170 cm; Chl *a* < 10 μg/L, measured according to EPA method 446, Arar and National Exposure Research Laboratory (U.S.), 1997). Double layers of nets (mesh size 1 cm) were fixed around the platform to exclude herbivorous fish.

### 2.2 Experimental design

**FIGURE 1:**
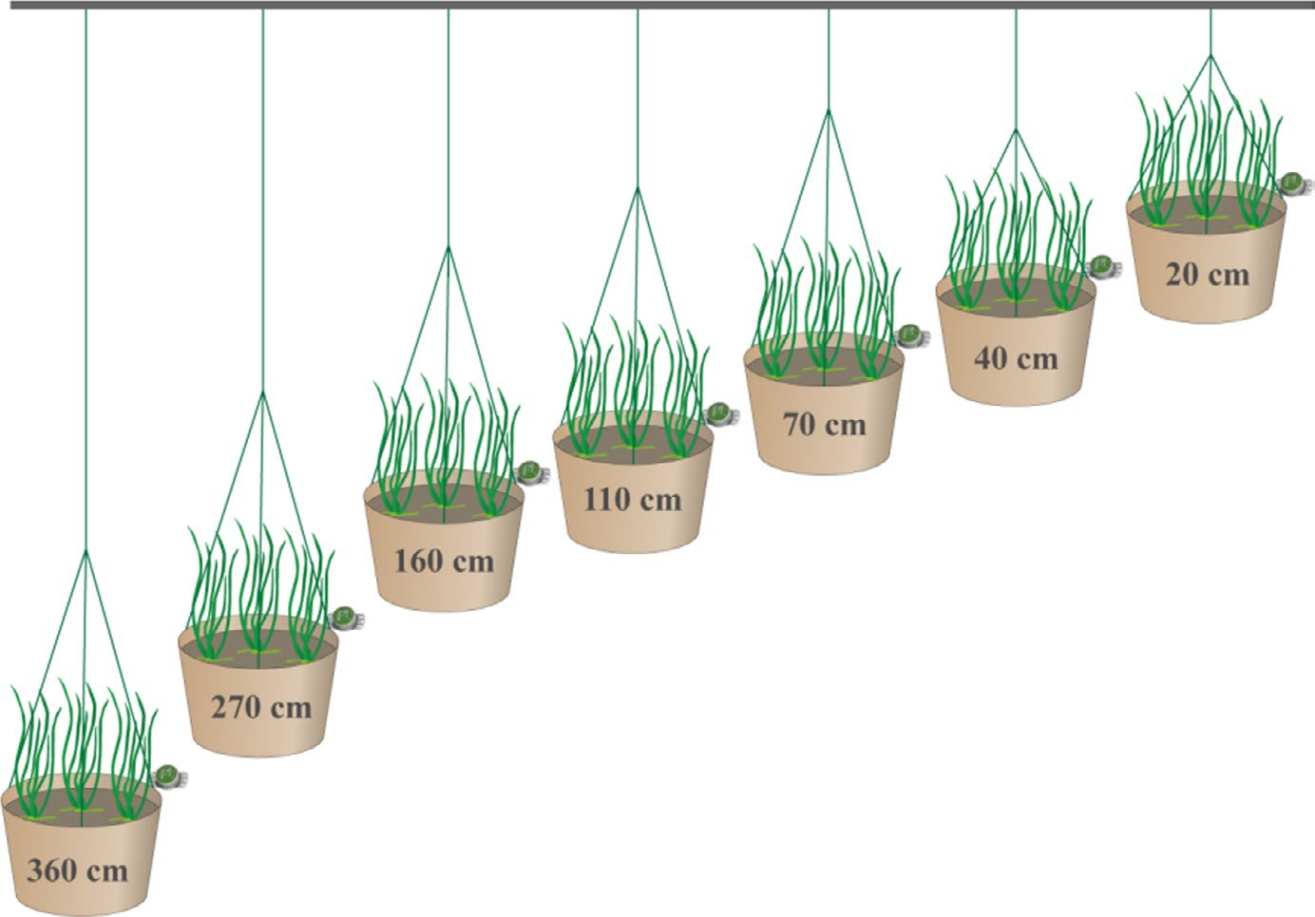
Layout of the in situ mesocosm experiment. Pots were hung at water depths of 20, 40, 70, 110, 160, 270, and 360 cm, arranged neatly and evenly along a high‐to‐low water depth gradient from south to north. *Vallisneria* images adapted from that created by Tracey Saxby, Integration and Application Network, University of Maryland Center for Environmental Science (http://ian.umces.edu/imagelibrary). No permission is required from the copyright holders for its reproduction

Shoots of *V. natans* used in this experiment were collected from Xukou Bay, a macrophyte‐dominated region located in the northeast of Taihu Lake (31°12′N, 120°28′E), as detailed in our former study (Wang, Gao, et al., 2021). To make sure that 4–6 shoots could survive and generate clonal juveniles, ten healthy shoots of *V. natans* with similar size (11.6 ± 1.2 cm height and 5 ± 1 leaves, mean ± *SD*) were planted evenly in one plastic pot (20 cm diameter, 15 cm height), containing 10‐cm‐deep argillaceous sediment (organic carbon 10.7 mg/g, total nitrogen: 0.87 mg/g, total phosphorus 0.08 mg/g). Before the initiation of the experiment, all these pots were pre‐incubated at 110 cm water depth for 14 days to facilitate generation of clonal population. Seven water depth treatments, that is, 20, 40, 70, 110, 160, 270, and 360 cm (Figure 1), in triplicate were set up on 3 June 2020. The experiment lasted for 4 weeks. The pots were arranged along a high‐to‐low water depth gradient from south to north and kept relatively steady throughout the experiment. To avoid shade, a distance of 50 cm was set between each pot and all pots were placed at least 50 cm away from the margins of the platform.

**FIGURE A2:**
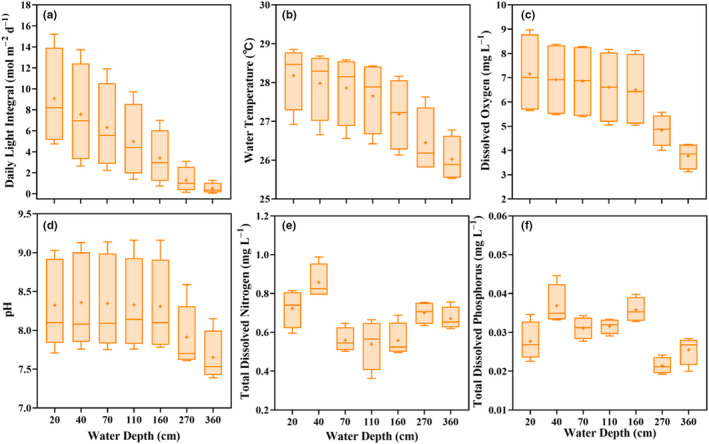
Boxplots showing the environmental factors measured at different water depths (20, 40, 70, 110, 160, 270, and 360 cm) during the period of the mesocosm experiment. (a) Daily light integral. The light intensity used to calculate the daily light integral was recorded at 2‐min intervals using a HOBO MX2202 logger (Onset Computer Corporation); (b) water temperature, recorded at 2‐min intervals using a HOBO MX2202 logger; (c) dissolved oxygen, measured using a probe (YSI ProPlus, Xylem Inc.); (d) pH, measured using a probe (YSI ProPlus, Xylem); (e) total dissolved nitrogen; (f) total dissolved phosphorus

During the experiment, light intensity and water temperature at each water depth were continuously measured at a 2‐min interval with the HOBO MX2202 Temp/Light data loggers (Onset Computer Corporation). In this study, we used the average daily light integral (DLI, mol photons m^−2^ d^−1^) as the quantifier of the light intensity experienced by the plants, since DLI rather than instantaneous light intensity relates better to the plants’ overall experienced light regime (Poorter et al., 2019). To calculate the DLI, the light measurements from the logger were converted to photosynthetically active radiation (PAR, measured in μmol photons m^−2^ s^−1^) based on a standard relationship (see Phillips, 2019; Thimijan & Heins, 1983), and the value of DLI was obtained by integrating the PAR over a day (Figure A2a,b). Water chemical parameters were measured at each depth every week. Specifically, pH and dissolved oxygen were measured with a portable water quality analyzer (YSI ProPlus, Xylem Inc.). Water samples from each depths were collected using a hydrophore and filtered with Whatman GF/F, and subsequently, total dissolved nitrogen and total dissolved phosphorus (Figure A2c,d) were determined photometrically (Ebina et al., 1983; Raveh & Avnimelech, 1979).

### 2.3 Traits measurements

**FIGURE A3:**
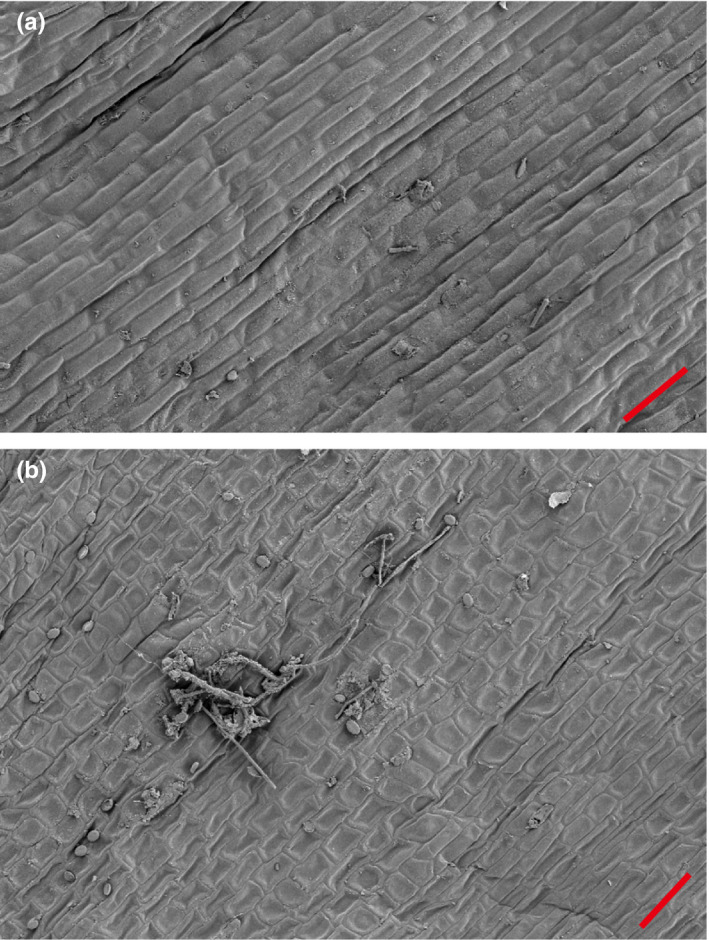
SEM micrograph showing the leaf epidermal cells of *Vallisneria natans* juveniles. Photographs were taken using a scanning electron microscope and morphological parameters were analyzed using the ImageJ software (https://imagej.nih.gov/ij/download.html); (a) 270 cm; (b) 20 cm; scale bar: 100 μm

At the end of the experiment, a range of plant traits (Table 1) of *V. natans* were measured. In brief, three *V. natans* juveniles (daughter ramets) from each pot were harvested, washed, and dried with filter paper to determine the total biomass, leaf number, leaf biomass, and leaf‐to‐total biomass ratio. In addition, leaf lamina length, leaf lamina width, and leaf thickness of the longest leaf of these juveniles were measured with a ruler (0.1 cm) and a digital micrometer caliper (0.001 mm). To calculate the specific leaf area and leaf dry matter content, ten intact leaves were randomly sampled from each pot. After scanning (Epson V39, Epson), the leaf area was calculated using ImageJ software. The specific leaf area was determined based on leaf area and the 60℃ dry weight (48 hr). Leaf dry matter content was determined based on the fresh weight and dry weight of the same leaves. For biochemical traits, the content of chlorophyll *a*, *b*, and carotenoids were, respectively, measured spectrophotometrically at 665, 649, and 470 nm using 95% ethanol as the solvent. Leaf soluble carbohydrate content, soluble protein content, peroxidase activity, and malonaldehyde content were determined using commercial reagent kits (Nanjing Jiancheng Bioengineering Institute, China, see details in Table A1). Following a scan with the electron microscope (GeminiSEM 300, Carl Zeiss), at least 50 cells per sample that were not a part of the midvein were measured with the ImageJ software to determine the leaf epidermal cell length and width (Figure A3).

**TABLE 1:** List of plant traits measured in the mesocosm experiment

No.	Full name	Abbr.	Explanations	Trait functions
1	Total biomass	TB	The fresh weight of an individual plant	Energy reserves
2	Leaf number	LN	Number of leaves per shoot	Light interception
3	Leaf biomass	LB	The total fresh weight of all leaves	Energy reserves
4	Leaf to total biomass ratio	L/TB	The ratio of leaves and shoot fresh weight	Responsiveness to light
5	Leaf dry matter content	LDMC	The ratio of leaf dry weight and fresh weight	Photosynthetic capacity, physical defense
6	Specific leaf area	SLA	The ratio of fresh leaf area and its dry weight	Photosynthetic capacity, physical defense
7	Soluble carbohydrate content	SC	Leaf soluble sugar content per unit fresh weight	Energy reserves
8	Soluble protein content	SP	Leaf soluble protein content per unit fresh weight	Energy reserves
9	Leaf lamina length	LL	Length of the longest leaf of an individual plant	Photosynthetic capacity, light interception
10	Leaf lamina width	LW	Width of the longest leaf of an individual plant	Photosynthetic capacity, light interception
11	Leaf lamina length‐to‐width ratio	L/W	The ratio of leaf length and leaf width	Responsiveness to light
12	Leaf thickness	LTH	Thickness of the longest leaf of an individual plant	Physical architecture
13	Leaf epidermal cell length	ECL	Average length of epidermal cells of the longest leaf	Light interception
14	Leaf epidermal cell width	ECW	Average width of epidermal cells of the longest leaf	Light interception
15	Epidermal cell length‐to‐width ratio	ECL/W	The ratio of epidermal cell length and epidermal cell width	Responsiveness to light
16	Chlorophyll *a* content	Chl *a*	Leaf chlorophyll *a* content per unit fresh weight	Photosynthetic capacity
17	Chlorophyll *b* content	Chl *b*	Leaf chlorophyll *b* content per unit fresh weight	Photosynthetic capacity
18	Chlorophyll *a* to *b* ratio	Chl *a*/*b*	The ratio of chlorophyll *a* content and chlorophyll *b* content	Responsiveness to light
19	Carotenoids content	Car	Leaf carotenoids content per unit fresh weight	Photoprotection
20	Carotenoids‐to‐chlorophyll ratio	Car/Chl	The ratio of carotenoids content and chlorophyll content	Photoprotection
21	Peroxidase activity	POD	Leaf peroxidase activity	Antioxidation capacity
22	Malonaldehyde content	MDA	Leaf malonaldehyde content	Oxidative damage

Note

Shaded cells were used to improve the readability.

### 2.4 Statistical analyses

All statistical analyses were performed in R version 4.0.3 (R Core Team, 2020) and SPSS 25.0 (SPSS, Inc.). To address the highly nonlinear and nonmonotonic relationships in ecological data, and without imposing any assumptions about the linearity of the relationship, we fitted generalized additive models (GAM) of the form *Y* ~ *s*(*X*) to analyze the effects of water depth on each plant trait. Models were built with the “gam” function (using thin plate regression splines) in the “mgcv” package, and number of knots (*k*, Table A2) was optimized manually based on the strength of the relationship (*R* ^2^). For GAM, a more conservative threshold of *p* < .001 was used (Woolway et al., 2021). The normality of the data was checked using Shapiro–Wilk's test. Redundancy analysis (RDA) was used to map the effects of environmental factors at different water depths on the measured plant traits of juvenile *V. natans*, using the “vegan” package. Response variables (plant traits excluded LN, SP, ECW, and Chl *a*/*b*) were centered and standardized before RDA. In order to distinguish the contribution of a single variable, the relative importance of each environmental factor independently accounting for the variations in plant traits was quantified by applying a hierarchy partitioning analysis, using the “rdacca.hp” package (Lai et al., 2021). Pearson's correlation analysis was performed in SPSS 25.0.

## 3 RESULTS

### 3.1 Variations in plant traits along a water depth gradient

**FIGURE 2:**
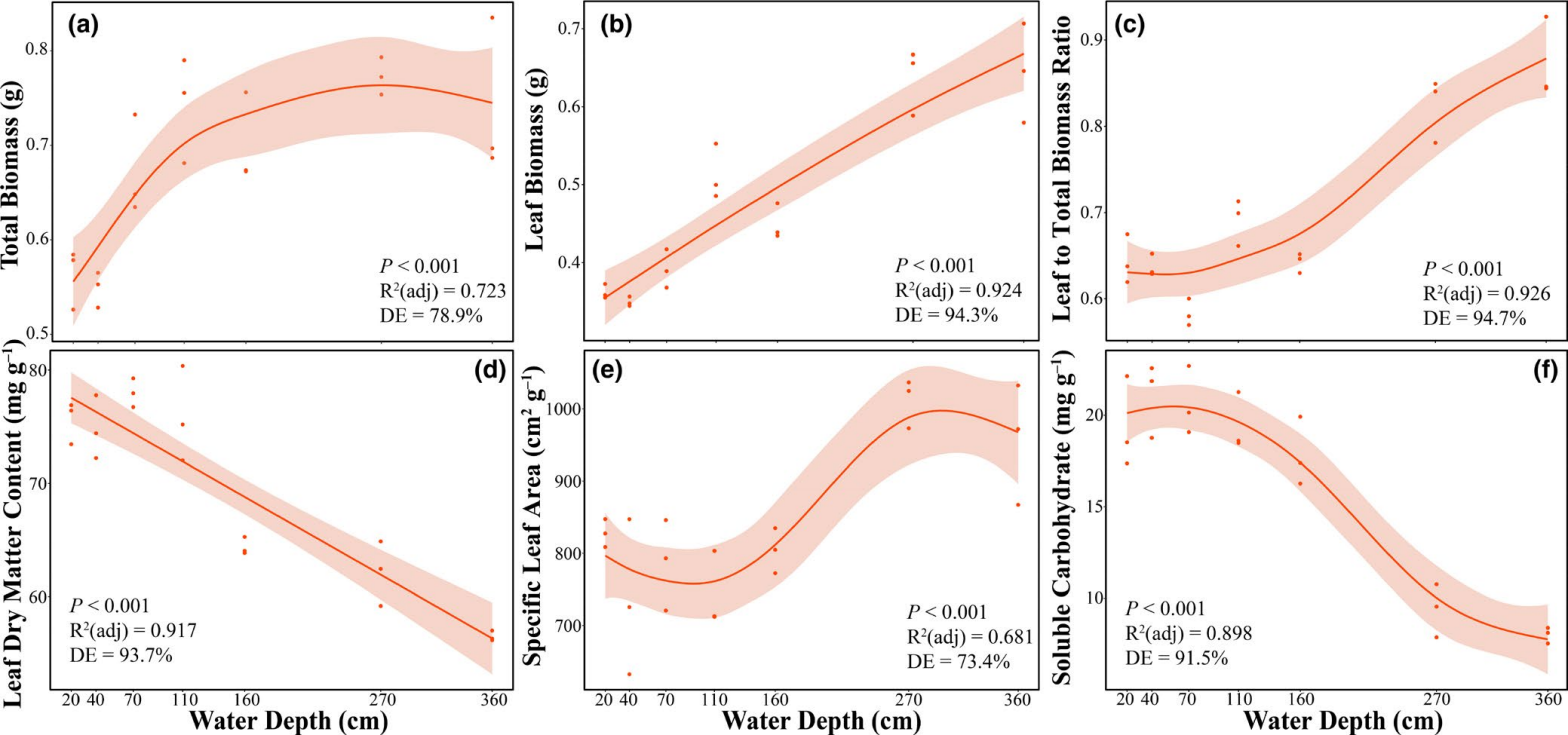
Growth and allocation traits (a–f) in dependence of the water depth gradient for *Vallisneria natans* juveniles. The gray shadow indicates a 95% confidential interval of the estimation. Representative parameters of GAM include the ability of regression equations to explain the variations (*R* ^2^ (adj)), the ability to interpret the overall changes of the variable (deviance explained, DE), and *p*‐values

**FIGURE A4:**
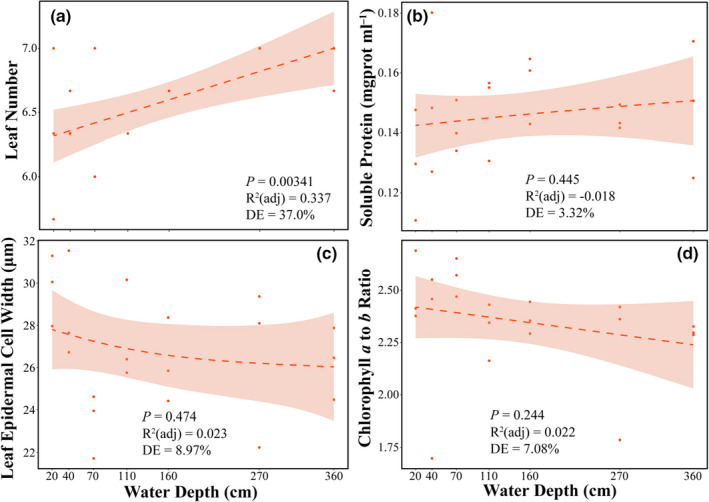
Plant traits (a–d) in dependence of the water depth gradient for *Vallisneria natans* juveniles. The gray shadow indicates a 95% confidential interval of the estimation. Dash line indicates no statistical significance (*p* > .001). Representative parameters of GAM include the ability of regression equations to explain the variations (*R* ^2^ (adj)), the ability to interpret the overall changes of the variable (deviance explained, DE), and *p*‐values. (a) contains identical values

Water depth exerted significant effects on most growth and allocation traits (Figure 2). A unimodal curve was found in the total biomass of *V. natans* juvenile (maximum at near 270 cm depth, Figure 2a). With increasing water depths, higher leaf biomass, leaf‐to‐total biomass ratio, but lower leaf dry matter content were found in *V. natans* juveniles (Figure 2b–d). In shallow waters, juvenile *V. natans* had higher leaf soluble carbohydrate content but lower specific leaf area (Figure 2e,f). Leaf number was, however, relatively stable with varying water depths (*p* > .001, Figure A4a), as well as the leaf soluble protein content (*p* = .445, Figure A4b).

**FIGURE 3:**
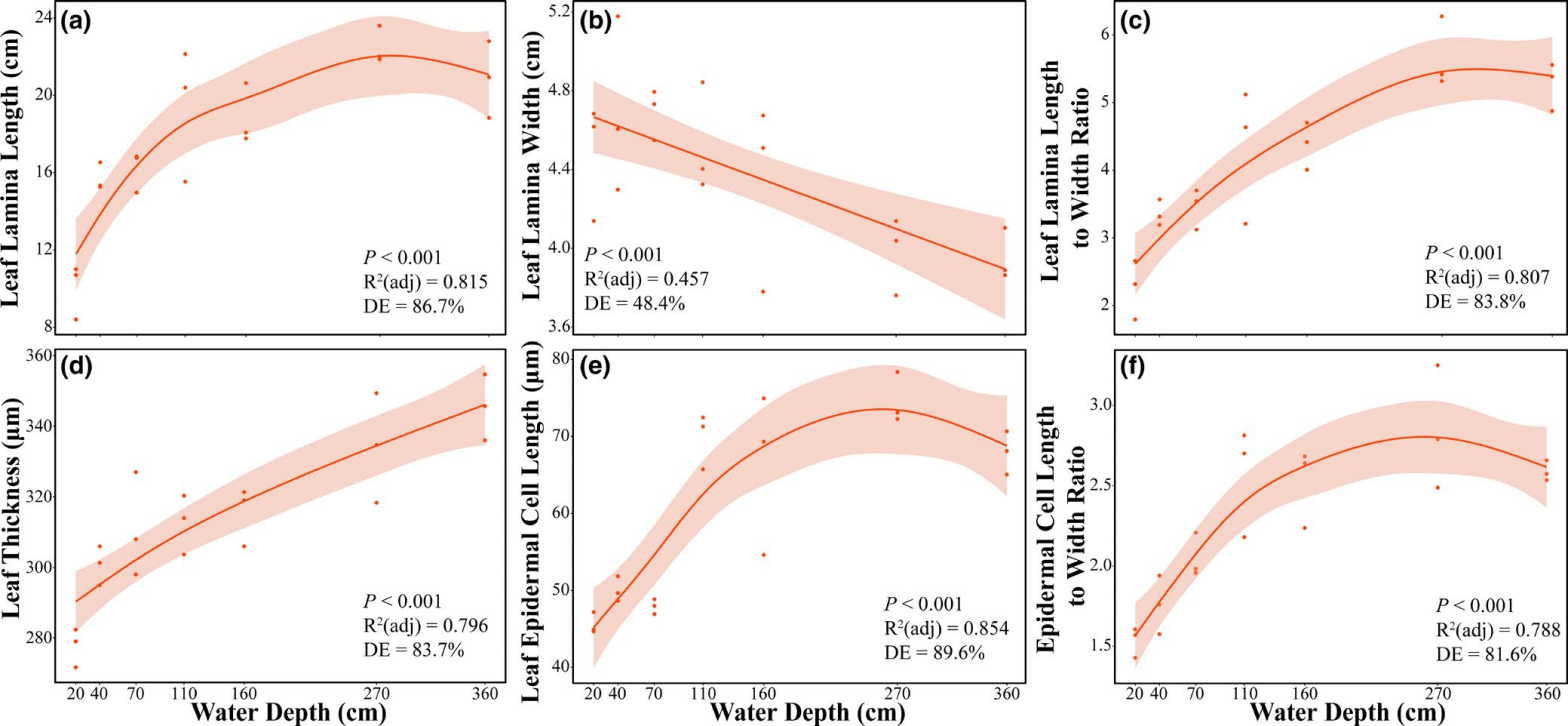
Leaf morphology and anatomy traits (a–f) in dependence of the water depth gradient for *Vallisneria natans* juveniles. The gray shadow indicates a 95% confidential interval of the estimation. Representative parameters of GAM include the ability of regression equations to explain the variations (*R* ^2^ (adj)), the ability to interpret the overall changes of the variable (deviance explained, DE), and *p*‐values

Leaf morphology and anatomy traits, except for leaf epidermal cell width, varied significantly along the water depth gradient (Figure 3; Figure A4c). Leaf lamina length, leaf epidermal cell length, and leaf epidermal cell length‐to‐width ratio of juvenile *V. natans* showed a unimodal curve, with the maximum at a near 270 cm water depth (Figure 3a,e,f). As for the leaf lamina width, a decreasing trend was found with increasing water depths, and correspondingly, the leaf lamina length‐to‐width ratio significantly increased with water depth (Figure 3b,c). Notably, thicker leaves of *V. natans* juveniles were observed with increasing water depths (Figure 3d).

**FIGURE 4:**
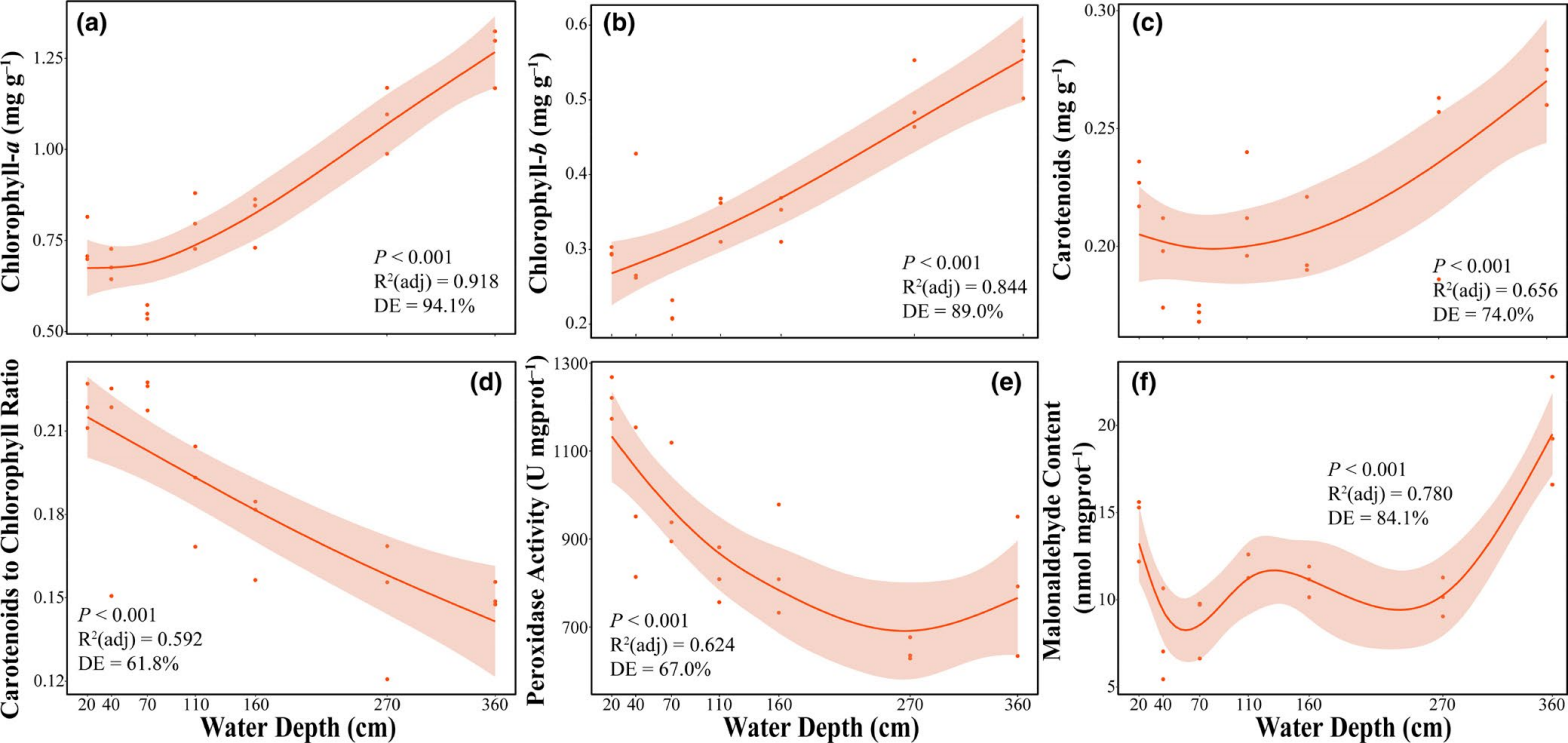
Leaf physiology traits (a–f) in dependence of the water depth gradient for *Vallisneria natans* juveniles. The gray shadow indicates a 95% confidential interval of the estimation. Representative parameters of GAM include the ability of regression equations to explain the variations (*R* ^2^ (adj)), the ability to interpret the overall changes of the variable (deviance explained, DE), and *p*‐values

Leaf physiological traits were significantly affected by water depth, with the exception of the chlorophyll *a* to *b* ratio (Figure 4; Figure A4d). As the water depth increased from 20 to 360 cm, concentrations of both chlorophylls (chl *a* and chl *b*) and carotenoids in leaves increased, while carotenoids‐to‐chlorophyll content ratio gradually decreased (Figure 4a–d). In addition, a decreasing peroxidase activity was found as water depth increased from 20 to 270 cm, followed by a slight increase at 360 cm, and the highest malonaldehyde content was observed at the 360 cm water depth (Figure 4d,f).

### 3.2 Relative effects of environmental factors on plants traits

**FIGURE 5:**
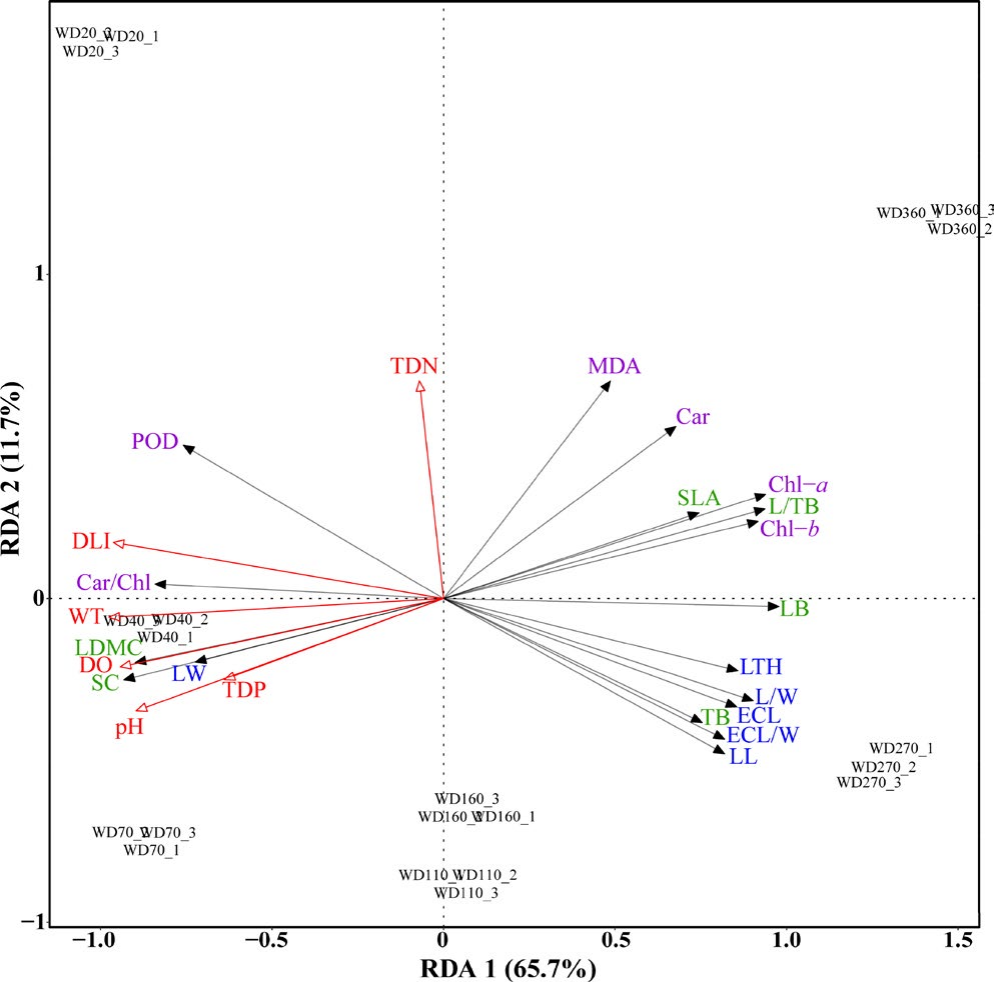
Redundancy analysis (RDA) plot showing the relationship between plant traits and environmental factors in the experiment. Environmental factors are indicated by the open‐headed red arrow pointing in the direction of increasing values, including DLI (daily light integral), WT (water temperature), DO (dissolved oxygen), pH, TDN (total dissolved nitrogen), and TDP (total dissolved phosphorus). Plant traits are indicated by solid‐headed black arrows pointing in the direction of increasing values. Traits of the same category are shown in the same color. The full names of abbreviated plant traits in this figure can be found in Table 1, and detailed RDA results refer to Table 2

Results of the redundancy analysis showed that six axes together accounted for 77.5% of the trait variation, among which the first two axes were overwhelmingly dominant (Table 2). The first RDA axis accounted for 66.7% of the trait variation and was mainly related to the covariation of water temperature and DLI, along the water depth gradient from deep to shallow water (Figure 5). The second RDA axis accounted for 11.8% of the variation and was related to the total dissolved nitrogen, which separated the extreme water depth (20 and 360 cm) from the other depths (40–270 cm).

**TABLE 2:** Redundancy analysis (RDA) of effects of different environmental factors on plant traits of *Vallisneria natans* juveniles

DCA < 3	RDA1	RDA2	RDA3	RDA4	RDA5	RDA6
Contribution to the variance						
Eigenvalue	11.8	2.12	0.465	0.320	0.230	0.198
Proportion Explained (%)	65.7	11.8	2.58	1.78	1.39	1.10
Cumulative Proportion (%)	65.7	77.5	80.1	81.8	83.2	84.3
Hierarchical Partitioning	DLI	WT	DO	pH	TDN	TDP
R^2^ (adj) = 0.673						
Independently Explained (%)	19.7	14.3	13.1	12.1	2.16	5.96

Note

As a preliminary step, detrended correspondence analysis (DCA) was used to validate the suitability of RDA. Environmental factors including DLI (daily light integral), WT (water temperature), DO (dissolved oxygen), pH, TDN (total dissolved nitrogen), and TDP (total dissolved phosphorus) were treated as explanatory variables, eighteen plant traits as response variables, for RDA. The relative importance of each explanatory variable independently accounting for the total variations was quantified by applying the hierarchy algorithm. Shaded cells were used to improve the readability.

Hierarchical positioning analysis demonstrated that DLI had the greatest effect on trait expressions among the six environmental factors (Table 2) included in this model (*R* ^2^ (adj) = 0.673): Positive correlations with DLI were observed for leaf dry matter content, soluble carbohydrate, carotenoids‐to‐chlorophyll content ratio, and leaf lamina width, while negative correlations were found for chlorophyll, specific leaf area, leaf biomass, leaf to total biomass, and most leaf morphology traits (Figure 5).

## 4 DISCUSSION

**FIGURE 6:**
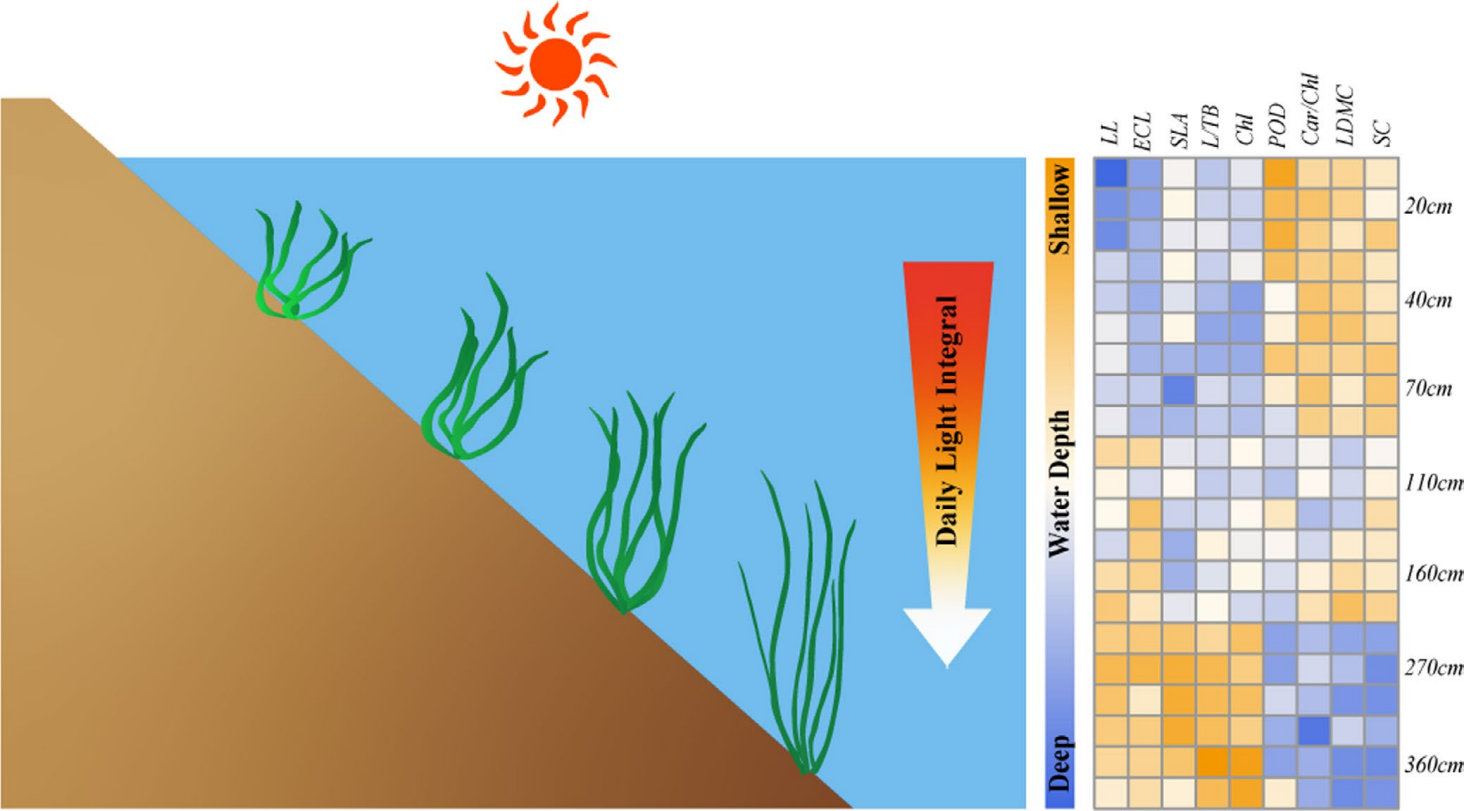
Schematic representation of main leaf‐related responses of *Vallisneria natans* juveniles along a water depth gradient. The heatmap was generated in R using "pheatmap" package. All three replicates were comprised in the heatmap. Deeper colors in the heatmap indicate higher (red) or lower (blue) values. *Vallisneria* images adapted from that created by Tracey Saxby, Integration and Application Network, University of Maryland Center for Environmental Science (http://ian.umces.edu/imagelibrary)

As we hypothesized, most plant traits of *V. natans* juveniles showed high plasticity in relation to water depth. Specifically, the trait syndrome (longer leaves, longer leaf epidermal cell, higher SLA, more biomass allocated to leaves, and higher Chl concentration) indicated greater resource acquisition with increased water depths. In shallow waters, however, the pattern of trait variations (higher LDMC, higher soluble carbohydrate, and more resources allocated to antioxidant protection) could be regarded as an indication of resource conservation (Figure 6). Despite the highly correlations among DLI, water temperature, and DO (Table A3), underwater DLI was found to be the most key factor driving the trait plasticity for *V. natans* juveniles based on the hierarchical positioning analysis.

In the present study, our results confirm regular changes in a suite of plant traits along increasing water depths. As stated in the balanced growth hypothesis, plant species preferentially allocate more biomass in the direction of the limiting resources (Shipley & Meziane, 2002). We found that juvenile *V. natans* tended to allocate more biomass to the leaves as water depth increased. Such results are in line with other results obtained from mesocosm experiments and field survey in Lake Erhai (Fu et al., 2012; Zhu et al., 2018). As for the rosette‐type macrophytes, the pronounced allocation pattern toward the leaf biomass benefits both light interception and light utilization by *V. natans* juveniles, indicating an adaptive response to the light attenuation in deep waters. In addition, changes in leaf economic traits also characterize the trade‐offs between investments and returns for nutrients (Reich & Cornelissen, 2014). According to the leaf economics spectrum (Pan et al., 2020; Wright et al., 2004), a higher SLA and lower LDMC of *V. natans* juveniles reflect fast resource acquisition and low resource conservation in deep waters. Moreover, leaf length and leaf width, respectively, increased and decreased as water depth rose, as well as a consequent increase in the leaf length‐to‐width ratio, indicating a trade‐off between light acquisition and leaf construction cost. Concomitantly, a similar trend was found in the leaf epidermal cell length as well as the ratio of cell length to width, which helps explain the variation in leaf morphology (Fricke et al., 1997).

In addition to growth/allocation traits and morphology/anatomy traits, leaf physiology traits of *V. natans* juveniles also adjusted in response to varying water depths. Consistent with the results of previous studies (e.g., Barko & Filbin, 1983; He et al., 2019), the content of leaf chlorophyll and carotenoids significantly increased with increasing water depths, which contributes to maintaining photosynthetic efficiency against the long‐term low light availability in deep waters. It is also worth noting that a relatively lower leaf POD activity was detected at water depth ≥270 cm. However, such finding is inconsistent with some previous studies, showing that increased water depth generally induces a significant enhancement in antioxidant enzyme activity (Li et al., 2020; Wang, Wang, et al., 2021). The enhanced enzyme activity could help scavenge the reactive oxygen species (ROS) generated under stress conditions, thus avoiding or reducing oxidative damage (García‐Caparrós et al., 2020). Therefore, our finding may imply that juvenile *V. natans* cannot adjust sufficiently to the 360 cm water depth, as the greatest MDA (product of lipid peroxidation) accumulation in *V. natans* leaves at such water depth, indicating an oxidative damage caused by excessive ROS. Interestingly, the highest total biomass in this study was found at around 270 cm depth (DLI: only 1.30 mol photons m^−2^ d^−1^). This characteristic would give this species a competitive advantage over other macrophytes under low light conditions. Together, our results verify the trait plasticity of *V. natans* juveniles in response to increasing water depths, which guarantees *V. natans* short‐term survival at a wide range of water depths.

In contrast, the traits of *V. natans* juveniles showed distinct characteristics in shallow waters. As a pivotal trait, the size of the mobile C‐pool (i.e., nonstructural carbohydrates, largely starch, and soluble carbohydrate) can mirror plants' carbon supply status (Würth et al., 2005). We found that the soluble carbohydrate content in *V. natans* leaves was highest at ≤70 cm water depths, and similar results were observed in an in situ experiment (Yuan et al., 2016), showing a significantly higher leaf soluble carbohydrate content for submerged macrophytes in shallow waters. On the one hand, the high metabolism level of plants in high light environments (shallow water) could lead to higher soluble carbohydrate contents (Sims & Pearcy, 1991). On the other hand, soluble carbohydrate may be tied to defense compounds (Würth et al., 2005), which partially explains its high correlation with LDMC (Palacio et al., 2008). Therefore, the high leaf soluble carbohydrate and LDMC contents in *V. natans* leaves reflect an optimization of resource conservation in shallow waters. Additionally, the ratio of carotenoids to chlorophyll (Car/Chl) was highest at 20–70 cm water depths, in line with a recent meta‐analysis study (Poorter et al., 2019). In view of the specific role of carotenoids in photoprotection (nonenzymatic antioxidants) and dissipating excess energy (xanthophyll cycle) (García‐Caparrós et al., 2020), the high Car/Chl ratio benefits plant leaves subjected to excessive light intensity. Similar to carotenoids, the POD also functions in photosystem protection by scavenging the ROS generated due to high light stress. In this study, the POD activity in *V. natans* leaves was highest at 20 cm water depth, also indicating a physical adjustment to intense light. Overall, our results suggest that, despite being a shade‐adapted species (light compensation point: 5.26–8.94 μmol photons m^−2^ s^−1^, see Ren et al., 1996), *V. natans* can adapt to shallow‐water environments through leaf trait plasticity at the juvenile stage.

Although reduced light intensity was found to be the key factor for the trait expression of *V. natans* juveniles (Harley & Findlay, 1994), this pattern of trait syndrome may also be induced by light quality, which was not measured in the present study. For shade‐avoiding terrestrial plants, recent studies have established that reductions in blue light, in addition to the drop in the R/FR ratio, lead to a shoot elongation and upward direction of leaves (Fiorucci & Fankhauser, 2017; Pedmale et al., 2016). While few studies have paid attention to the effects of light quality on macrophytes (Momokawa et al., 2011), in inland waters with high concentrations of chromophoric dissolved organic matter, blue light may serve as such a cue because blue light diminishes with increasing water depths (Kirk, 2011). Thus, future studies are needed to examine the role of underwater light quality in explaining the adaptive responses of submerged macrophytes to varying water depth, as well as the field studies performed in lakes with different characteristics in underwater light field. In addition, attentions must be paid to other depth‐related factors such as water temperature and DO. Varying water temperature and DO naturally could result from the variations in DLI. Therefore, further studies should isolate a single variable from the others to discern the specific responses of macrophytes and the related ecological consequences.

## 5 CONCLUSIONS

In the present study, we examined 22 leaf‐related traits of *V. natans* juveniles grown at different water depths in an in situ mesocosm experiment. Our results clearly demonstrate that juveniles of *V. natans* can rapidly respond to the water depth‐induced variation in light intensity through leaf trait plasticity, thus adjusting to a wide range of water depths in a short term. Particularly, the trait syndrome of juveniles grown in deep waters represents an optimization of resource acquisition, while it shifts toward resource conservation in the shallow waters. Based on these knowledge, the present study can help explain why *Vallisneria* spp. often dominates in field plant communities in relatively deep waters (He et al., 2019; Sheldon & Boylen, 1977), but has a decreased competitive advantage in shallow waters (Li et al., 2019).

## CONFLICT OF INTEREST

The authors declare that they have no conflict of interest.

## AUTHOR CONTRIBUTIONS

**Yuxuan Gao:** Formal analysis (supporting); Investigation (supporting); Visualization (lead); Writing‐original draft (equal); Writing‐review & editing (equal). **Lei Wang:** Formal analysis (lead); Investigation (lead); Visualization (supporting); Writing‐original draft (supporting); Writing‐review & editing (supporting). **Xiaoqing Hu:** Investigation (supporting). **Zhuolun Zhang:** Investigation (supporting). **Baogui Liu:** Investigation (supporting); Supervision (equal). **Xinhou Zhang:** Supervision (equal); Writing‐original draft (equal); Writing‐review & editing (equal). **Guoxiang Wang:** Conceptualization (lead); Funding acquisition (lead); Supervision (equal).

## Data Availability

The dataset used for analysis has been archived in Dryad Digital Repository https://doi.org/10.5061/dryad.1g1jwstx5.

## APPENDIX A

**TABLE A1:** Details of the commercial reagent kits used in this study

Full name	Abbr.	Method	Absorption peak	Appendix Reference
Soluble carbohydrate content	SC	Anthrone colorimetry	620 nm	Yemm and Willis (1954)
Soluble protein content	SP	Bradford assay	595 nm	Bradford (1976)
Peroxidase activity	POD	Guaiacol oxidation method	470 nm	Chance and Maehly (1995)
Malonaldehyde content	MDA	TBA method	532 nm	Hodges et al. (1999)

**TABLE A2:** Parameters of the GAMs

No.	Full name	Abbr.	Regression smoother	Number of knots
1	Total biomass	TB	s	7
2	Leaf number	LN	s	7
3	Leaf biomass	LB	s	6
4	Leaf to total biomass ratio	L/TB	s	7
5	Leaf dry matter content	LDMC	s	6
6	Specific leaf area	SLA	s	7
7	Soluble carbohydrate content	SC	s	7
8	Soluble protein content	SP	s	7
9	Leaf lamina length	LL	s	7
10	Leaf lamina width	LW	s	7
11	Leaf lamina length to width ratio	L/W	s	5
12	Leaf thickness	LTH	s	7
13	Leaf epidermal cell length	ECL	s	7
14	Leaf epidermal cell width	ECW	s	7
15	Epidermal cell length to width ratio	ECL/W	s	7
16	Chlorophyll *a* content	Chl‐*a*	s	7
17	Chlorophyll *b* content	Chl‐*b*	s	7
18	Chlorophyll *a* to *b* ratio	Chl *a*/*b*	s	7
19	Carotenoids content	Car	s	6
20	Carotenoids to chlorophyll ratio	Car/Chl	s	7
21	Peroxidase activity	POD	s	7
22	Malonaldehyde content	MDA	s	7

**TABLE A3:** Results of Pearson's correlation analysis among the abiotic variables

	DLI	WT	DO	pH	TDN	TDP
DLI	1					
WT	0.969**	1				
DO	0.896**	0.970**	1			
pH	0.824*	0.932**	0.988**	1		
TDN	0.267	0.054	−0.131	−0.236	1	
TDP	0.523	0.608	0.645	0.687	−0.185	1

**p* < .05, ***p* < .01.
